# Sharing the Field: Reflections of More-Than-Human Field/work Encounters

**DOI:** 10.1080/2373566X.2021.2016467

**Published:** 2022-02-08

**Authors:** Natalie Marr, Mirjami Lantto, Maia Larsen, Kate Judith, Sage Brice, Jessica Phoenix, Catherine Oliver, Olivia Mason, Sarah Thomas

**Affiliations:** University of Glasgow, UK; University of New South Wales, Australia; University of Bristol, UK; Lancaster University, UK; University of Birmingham, UK; Durham University, UK

**Keywords:** encounter, feminist epistemologies, fieldwork, knowledge practices, multispecies relations, 接触&#, 女权主义认识论&#, 实地考察&#, 知识实践&#, 多物种关系, encuentro, epistemologías feministas, prácticas de conocimiento, relaciones entre especies, trabajo de campo

## Abstract

The “field” has long been contested as spatially and temporally bounded. Feminist epistemologies have re-imagined and engaged field/work as shared, messy and co-constitutive, while critical more-than-human methodologies in the transdisciplinary field of the environmental humanities are further expanding our understanding of who and what counts in the production of knowledge in the field. This compendium article orbits around a collective concern for the sharedness of bodily and planetary ecologies through field/work. It brings together cross-disciplinary accounts of field encounters that critically explore what it feels like to do this work and what it entails. With a focus on practice and process, the six contributing authors—researchers, artists, practitioners, writers—consider how nonhumans share in our research, shaping the work we do, the questions we ask and the responses we craft. Together, they offer thoughtful provocations on the troubling and promising ways in which human and non-human bodies become unsettled and rearranged through field encounters.

## Abstract (zh)

“实地”在空间和时间上一直都有争议。女权主义认识论将实地&#考察&#重新假想为共享、混乱和共同构成。在跨学科的环境人文学领域中&#，批判性的超人类方法论&#，进一步拓展了我们对谁和什么在知识生产中发挥作用的理解。该概要文章围绕了一个共同关注点—基于实地&#（考察&#）的共享式身体生态和地球生态。它汇集了对实地接触的跨学科描述&#，批判性地探索了从事这项工作的感受和所需要做的工作。本文注重实践和过程&#，六位作者&#（研究人员、艺术家、实践人员、作家&#）考虑了各种非人类如何在研究中进行共享、如何塑造了我们的工作、问题和回应。一些令人不安但有希望的方式&#，能使得人类和非人类在实地接触中变得不稳定或进行重组&#，作者对这些方式提出了深思熟虑的挑战。

## Abstract (es)

Desde hace mucho tiempo se cuestiona el “campo” como concepto delimitado espacial y temporalmente. Las epistemologías feministas han re-imaginado y comprometido el trabajo de campo como algo compartido, desordenado y co-constitutivo, en tanto que las metodologías críticas más-que-humanas en el campo transdisciplinario de las humanidades ambientales están ampliando aún más nuestro entendimiento sobre quién y qué cuenta en la producción de conocimiento en el campo. Este artículo de compendio gira alrededor de una preocupación colectiva de compartir las ecologías corporales y planetarias a través del trabajo de campo. El artículo reúne los relatos interdisciplinarios de encuentros en el campo que exploran críticamente lo que se siente al ejecutar este trabajo y lo que éste implica. Enfocándose en la práctica y en el proceso, los seis autores que colaboran ––investigadores, artistas, profesionales, escritores–– consideran cómo los no humanos comparten en nuestra investigación, dándole forma al trabajo que hacemos, a las preguntas que formulamos y a las respuestas que conseguimos. Juntos, ellos ofrecen reflexivas provocaciones sobre las formas inquietantes y promisoras en las que los cuerpos humanos y no humanos se desestabilizan y se reorganizan a través de encuentros en el campo.

## FIELDS SHARED AND REARRANGED: AN INTRODUCTION

### Natalie Marr, Mirjami Lantto, and Maia Larsen, University of Glasgow, UK

This compendium article orbits around a collective concern for the *sharedness* of bodily and planetary ecologies, reflecting on the troubling and promising ways in which human and non-human bodies become unsettled and rearranged through (field) encounters. The pieces have come together from a double conference session at the 2019 RGS-IBG Annual Conference at the Royal Geographical Society in London. The session—*Visitations: more-than-human field/work encounters*—invited speakers to share reflective accounts of fieldwork with nonhumans. Focusing on process and practice, we asked: what does it *feel* like to do this work and what does this entail? How do our respective “fields” shape the work we do and the questions we ask?

As a “distinct moment” in research (Massey 2003), the field has long been contested as spatially and temporally bounded, in which field encounters are subject to a masculinist, colonialist logic of exploration and extraction (Hyndman 2001; Katz 1994; Rose 1997; Sundberg 2014). Feminist epistemologies have instead explored our worldly engagements in terms of messy transcorporealities (Alaimo 2010) and “reciprocal capture” (Stengers 2010), weaving an imagination of fieldwork as “withness” (Volvey 2012), less a foreclosed space-time of activity and more an engaged practice, receptive to what transpires. The development of critical more-than-human methodologies and critical place enquiry in the transdisciplinary field of environmental humanities is further expanding our understanding of who and what counts in the production of knowledge in the field (Bastian et al. 2017; Thomas 2015; Tuck and McKenzie 2015). This work shows how nonhumans *share in* our research processes and practices, co-composing and rearranging our fields. It also raises important questions and challenges regarding how researchers engage modes of enquiry that are cognizant of and responsive to other epistemic worlds (Sundberg 2014).

To *field* is to attempt to catch an object and return it; to grapple with a difficult situation, a challenging question or predicament. In *fielding* we are called to engage with what presents itself in the moment, *in situ*. Cast in this light, the researcher is not a stable figure, able to enter and exit the field untouched, but rather one element within a shared ecology, involved in a relationship of practice that is negotiated and co-constitutive (Alaimo 2010; Billo and Hiemstra 2013; Rose 1997).

To share is both to hold in common and to be divided. The English word “share” links back to the Old English *scearu* and *scær*, and the Old Frisian *skere*: a cutting, shearing; a part or division; a thing that cuts (Share n.d.). This evokes both the rupture and rapture of more-than-human (field) encounters; how fieldwork is both a cut in the world (Candea 2007) and the experience of the world as it disorders and rearranges our work and selves (Tsing et al. 2017; Wilson 2017). As the pieces below will demonstrate, the experience of sharedness through fieldwork is not a seamless merging of researcher and site, but often uncertain, fraught and unsettling. Sharedness is risky, exposing and uneven. It requires ways of being in and *with* the field that incorporate acts of listening and witnessing (Boscacci 2018; Kanngieser 2020, 2015). It asks that we develop the capacity to respond. Fielding, then, requires a commitment to the difficult and thorny experience of sharedness; to “take up the unasked-for obligations of having met” (Haraway 2016, 130).

In the first two pieces, the composition, trans-corporeality and co-becoming of human and non-human bodies emerge as key considerations for the murky matters of shared ecologies. Tracing the movement of the Ross River virus through different bodies in the mangroves of Sydney’s Georges River, Kate Judith discusses the “ancient and immediate,” less-than harmonious ways in which bodies attune to one another. Her storying through these attunements troubles the singularity of bodies, evoking uncertainty about “just who is becoming entangled and what motivations are involved.” With a shared concern for porous corporealities, Sage Brice approaches bodies as “provisional constellations” through the practice of drawing Eurasian cranes in the Huleh valley in northern Israel-Palestine. Committed to the vulnerable and fleeting composition of bodies in the field, she discusses how a desire to “fix” the cranes is re-oriented to an appreciation of “how and why they might elude capture.”

In the subsequent three pieces, *sharedness* is made manifest in the particularities of violence and care present in our fraught relations with non-human others. Our attention is drawn to the shifting nature of these relations in Jessica Phoenix’s reflection on her conflicting encounters with badgers as she follows bovine Tuberculosis (bTB) across England. In negotiating moments of violence, care and disconnect across the different “wheres” and “whens” of fieldwork, her relations with badgers become situated in the complex geographies of multispecies encounters as they refract through one another. Catherine Oliver discusses parallel concerns in a narration of her relations with ex-battery hens, considering the ways in which pain and mourning become distributed across bodies. Seeking a multispecies ethics and politics of care, she calls for a radical commitment to uncertainty as an “intentional interruption of the violent human-as-usual,” re-orienting us toward more hopeful multispecies futures. The unevenness of our shared realities with nonhuman others is further explored by Olivia Mason, through an attendance to the shifting relations between humans and donkeys in Petra, Jordan. Through a focus on differing moments of touch, her discussion reveals how multispecies relations are entangled with a story of Bedouin displacement.

The article is drawn to a tentative close with Sarah Thomas’s reflection on her experience of writing-with a landscape and community unsettled by a flood in Cumbria. The piece contemplates what happens when the field is also home, and “the vulnerability of the researcher is not only a source of theoretical consideration but a pressing reality,” pulling us into sensations of immediacy *and* estrangement that weave the fabric of our shared lives. Speaking to Oliver’s call for a committed engagement with uncertain futures, Thomas grapples with *sharedness* not only as collective vulnerability, but as the crafting of meaningful response.

## THE WALLABY, THE MOSQUITO, THE VIRUS AND ME

### Kate Judith, University of New South Wales, Australia

For the last three years I have been exploring interstitiality with the help of the mangroves of Sydney’s Georges River. I live not far from these mangroves and visit them often, reading and writing there, thinking through issues and concepts, or just staring out through the twisted boughs. My research and mangrove materialities are enfolded in many ways, including theoretically and viscerally. Here I follow one such enfolding as research field and researcher’s body entangle in problematic relationships of co-becoming that are both ancient and immediate.

To the landward side of these mangroves, protected from tidal disturbances, brackish water forms stagnant pools where female saltmarsh mosquitos, *Aedes vigilax*, lay their eggs. These mosquitoes, which are the main vector for Ross River virus (RRV) through Australia are abundant in the Georges River region during the summer. RRV infections can cause flu-like pain, fatigue and rash lasting from a few weeks to several years (Claflin and Webb 2015; Harley, Sleigh, and Ritchie 2001). In this region wallabies are the main reservoir hosts (Stephenson et al. 2018). Studies on horses and lab mice find they suffer a range of RRV symptoms including swollen joints and lethargy (El-Hage, McCluskey, and Azuolas 2008; Redfern 2015), so perhaps it is a stiff and weary wallaby who ventures out at dusk into a grassy clearing near the river, for RVV has been multiplying rapidly within her blood for the past few days. Although the virus may have been present for some time, it is only abundant enough within her blood to be picked up by a mosquito during a one to six day period (Harley, Sleigh, and Ritchie 2001, 118). Today, huge numbers of viral envelopes are circulating within this wallaby’s blood.

This same evening, a female *saltmarsh* mosquito launches out from the underside of the leaf protecting her from the summer sun. She is hungry for animal blood because she is pregnant and her eggs require blood for their development. The blood will only nourish her eggs, the mosquito sups nectar for her own energy. Already we cannot tell whose hunger this mosquito is. Is her hunger her own, or does it only call down through her cells from a future to live into its already diverging forms? Perhaps it includes the hunger of her microbiotic guests demanding sustenance. These include long term bacterial and protozoan symbiotic residents who contribute digestive products throughout her body and interact with viruses there (Novakova et al. 2017). Our relationships are beginning, but it is uncertain just who is becoming entangled and what motivations are involved. This hunger piques the sensitivity of mosquito scent receptors which discriminate favored warm animals by the particular mix of their many different odors. She moves into the breeze toward them, honing in further when she sees the shape and size of a wallaby and closer still as she detects the heat from wallaby skin (Stoller-Conrad 2015). When the mosquito bites she drinks up a good dose of RRV.

To move through the mosquito the virus must negotiate the barriers and challenges of her body. First it must infect and replicate inside her midgut (Rückert and Ebel 2018) where bacteria such as *Wolbachia* is likely to diminish successful virus reproduction^1^ (Kerney et al. 2017). Once through the gut barrier the virus encounters the mosquito’s immune system as it infects body cells. Passing through a final barrier into the salivary glands, the virus replicates quickly and waits.

It’s a warm summer evening and I’m at my desk, a couple of kilometers from the Georges River. My ears are receiving many sounds but none disturb me until I hear a delicate, high pitched buzz. I stiffen and grow alert, my eyes darting around the room searching for a fragile, black creature whose plans I would destroy with repellent or a slap. When she lands on my ankle, her long lithe legs spread her weight so the sensitive nerves near my skin’s surface do not detect her. Her mosquito proboscis is a highly specialized mouth comprising six separate needlelike stylets (Quirós 2016). Two of these have cutting teeth to pierce through the skin and another two hold it open. Saliva, containing substances that thin my blood, is injected through the fifth and the sixth is the tube which will carry my blood back to the mosquito’s digestive system. She delicately slices into the skin and searches nimbly through the cells, following the taste of blood. When my ankle itches I feel she has outwitted me. As I scratch, the vague fear of RRV crosses my mind, in the form of the memory of a map of its prevalence around this region.

As saliva from the mosquito seeps around my skin cells, a wide range of immune cells begin responding (Briant et al. 2014; Redfern 2015). Some move to the feeding site to signal further immune processes. Others release compounds that cause inflammatory responses. The virus traveling with the saliva also quickly triggers my immune system (Redfern 2015). Within some skin cells, viruses similar to RRV stimulate strong immune responses and fail to replicate and spread, but in others, such as the large Langerhans cells which move around through the skin layers, these viruses can move through the skin layers to other parts of my body (Briant et al. 2014, 27).

Most of the time RRV exists as a single strand of RNA 11,853 nucleotides long which includes codes for four proteins. One of these proteins forms a viral coat, another forms a lipid layer around the coat and the remaining two protrude from the surface (Harley, Sleigh, and Ritchie 2001, 911). When a viral envelope contacts one of my cell walls, one of these proteins reduces the PH of the cell surface allowing the virus code to enter. If not disabled by immune factors, once inside my cell the viral genome dampens the normal work of the cell, while interacting with the cell’s components to reproduce and reassemble viral proteins (Redfern 2015, 10–2). Somewhere between 40 and 80% of the human genome may be of ancient viral origin that integrated into host DNA (Letzter 2018; Parrish and Tomonaga 2016). This viral-origin genetic material plays a role in immune system and placental functioning, and during early fetal development. It is also important in brain functioning where it may explain variations in adult cognitive function (de La Torre-ubieta et al. 2018) and important aspects of brain evolution (Briggs et al. 2015).

Over thousands of generations, mammal and mosquito bodies, viruses and immune systems, have responded to each other to the point where separating our parts becomes difficult. My hearing is primed to the mosquito’s faint whine. The mosquito is attuned to the chemicals exhaled from my body. My skin’s immunological capacity is prepared to receive her entry. Her mouth is able to pierce and search, her eggs are ready to be nourished with my blood. We are continually attuning, but none of us is oriented toward the welfare of the others, nor even to further the welfare of us as an entangled being. We are ostensibly harmful to each other. Attunement does not come from a desire for harmony, stability or sustainability. It continues as each responds to the opportunities and irritations proposed by the others. Ongoing agonistic negotiations tangle pain and illness with nourishment and adaptive vigor. We keep pricking each other into our futures within a field located within and between our bodies.

## DRAWING COMPOSITE BODIES: REFLECTIONS ON REPRESENTATION AND VULNERABILITY IN GEOGRAPHICAL FIELDWORK

### Sage Brice, University of Bristol, UK

“Why should our bodies end at the skin, or include at best other beings encapsulated by skin?” (Haraway 1991, 178).

A world alive moves too quick to catch. This is a frustration of drawing nonhuman animals in the field, but it is also an interesting problem to sit with. After all, “capturing” or “fixing” nonhuman bodies is a less interesting research goal than appreciating how and why they might elude capture. Like the wider traditions of “natural history” within which they take shape, practices of observational field drawing have historically been bound up with colonial logics of acquisition and classification, though they have also been celebrated for the more open and transformative possibilities they may engender (Brice 2018; Causey 2017; Foster and Lorimer 2007; Ingold 2011; Kuschnir 2016; Parikh 2019).

**FIGURE 1:**
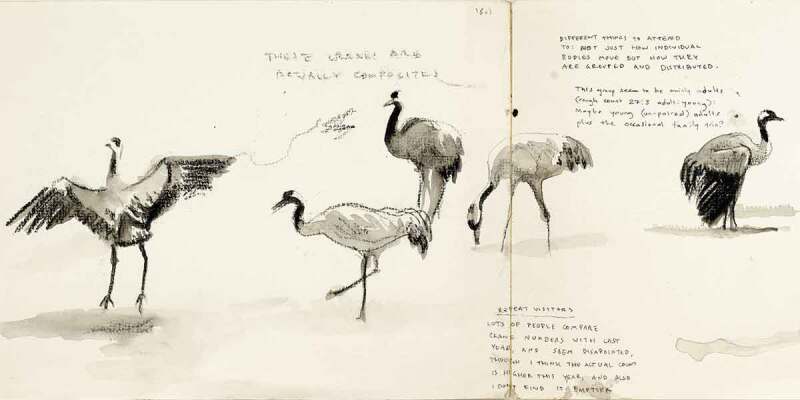
“These cranes are actually composites” (Field journal, Huleh Valley, 14 January 2017). Image by Sage Brice.

**FIGURE 2:**
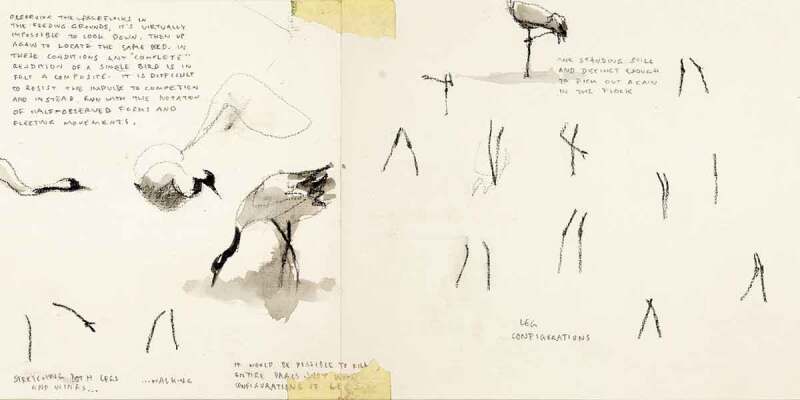
Composite birds and leg configurations (Field journal, Huleh Valley, 27 February 2017). Image by Sage Brice.

Research in cultural geography and its cognate fields has in recent years increasingly drawn attention to the ways in which individual bodies, while important spatial sites in their own right, emerge only as provisional constellations of relations within wider ecologies of bodies and ideas (Haraway 1991; Manning 2013; McCormack 2013; Tsing et al. 2017). Such work echoes much indigenous knowledge and scholarship in that it refutes the ontological priority of individual (id)entities (Barker and Pickerill 2020; Escobar 1996; Little Bear 2000). A wealth of scholarship has addressed the ways in which this refutation also raises questions about the role of representation (which presumes a prior “given-ness” of observable entities) in processes of knowledge generation (Anderson and Harrison 2010; Barad 2007; Colls 2012; Dewsbury 2012; Lorimer 2005; Thrift 2008). The above excerpts from my field journal (Figures 1 and 2) document two observations in a practice-led process, in which these questions were made present through the methodological particularities of my research. The research project in question explored drawing as a mode of practicing vulnerability in geographical fieldwork. Observational drawing was my primary method for studying encounters between humans (*H. sapiens*) and Eurasian cranes (*G. grus*) in the Huleh Valley in northern Israel-Palestine. The project explored a number of senses in which vulnerability—understood as an openness to the permeability and mutual constitution of bodies and ideas—is made manifest through the practice of drawing as fieldwork (Brice 2018, 2020a, 2021).

This short piece outlines a very practical sense in which drawing attuned me to the permeability of animal bodies. The drawings reproduced above show two moments at which I observed that the crane forms I was drawing on the page were, in fact, composites of a number of birds, picked out from among a crowd numbering tens of thousands of cranes. As I drew, my gaze flicked back and forth between the page and the birds, sometimes hampered also by unwieldy binoculars. I could take in only multiple, fleeting impressions of bodies that constantly rearranged and composed themselves. The attempt to reconcile these impressions into coherent forms on the page was, in one sense, a representational conceit that belied the complexity and movement I was confronted with. At the same time, I was interested less in the production of images themselves than in what I could learn from the process of trying to draw them. The struggle involved in that process gave me an opportunity to stay close to the problems of representation. The impact of this practice was cumulative and iterative: through repeatedly attempting to integrate multiple fleeting impressions I was able to slowly acquire an understanding of crane form and movement in a way I could not have done without that discipline. At the same time, the repeated process kept me always cognizant of the apparent sleight of hand necessarily involved in the production of notional, fixed entities. I could not have been more viscerally aware that these bodies were composite—not only as drawings, but in relation to and with one another and with the wider ecology in which they participated. That is an important sense in which drawing in this project served as a mode of vulnerability. It helped destabilize any tendency to think of bodies in the field—my own, and those of the birds and other humans I observed—as fixed or coherent entities.

In this way, observational drawing offered a mode of “vulnerable” enquiry through which to develop an account of the material, affective, and ideal ecologies of the Huleh Valley that was not predicated upon the presumed coherence of individual bodies, or of distinct identity groups such as species, gender, or nation. Instead, it enabled me to attend to the movement and distribution of relational affects across such lines of categorical difference. The resulting thesis argued that cranes are actively implicated and involved in the formation of a gendered politics of national identity and belonging in the Huleh Valley (Brice 2020b). More specifically, drawing allowed me to explore the ways in which images and stories can be observed to exceed logics of representation (Anderson 2019; Keating 2019). In this way, the project drew together the two theoretical imperatives indicated above: understanding individual entities as constellations of relations within a wider ecology, and moving beyond representational logics which assume the “given-ness” of such entities.

## WHERE AND HOW SPECIES MEET MATTERS: BADGERS, BOVINE TUBERCULOSIS AND TROUBLING ENCOUNTERS

### Jessica Phoenix, Lancaster University, UK

[Content warning: the following piece contains images of animal death].

Should wild badgers be culled to control the spread of the disease bovine Tuberculosis (bTB) in cattle in England? First asked in the 1970s, the ensuing debate resulted in badger culling being instigated in parts of England from 2013. The cull has raised impassioned contention between those for and against the practice, which shows no sign of resolution.

In 2016–2018 I followed bTB across England, working closely with badgers throughout. I met badgers in multiple places and through multiple practices, and my relationship to them was in constant flux. In this piece I provide reflexive accounts of my relations with badgers using fieldnotes and photographs from participant observation of four encounters.

I use these encounters to empirically investigate *how* and *where* species meet matters. Building on Haraway (2008) and Hinchliffe (2010), I suggest that individual encounters with more-than-humans across multiple practices and in multiple sites can form thoughtful relations with species as a collective.

### Shooting Badgers

**FIGURE 3:**
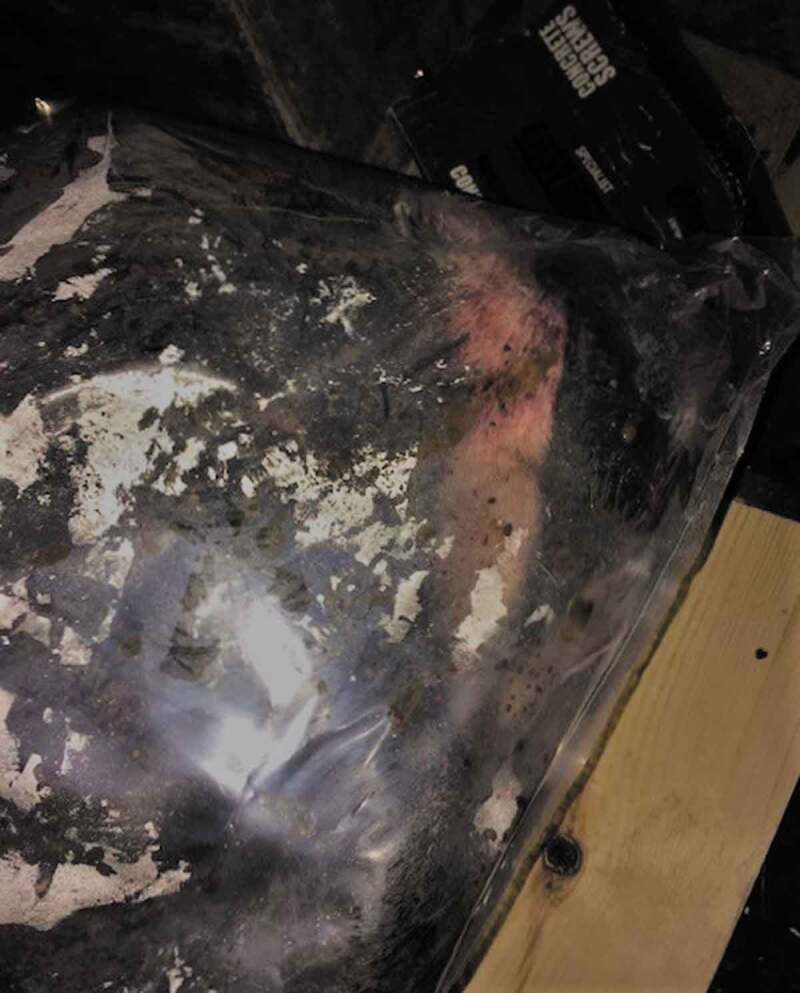
Shot badger in a double bag ready for disposal. Photo by Jessica Phoenix.

I spent last night with George and Fred.^2^ At approx. 00:15 Fred stopped dead in his tracks because he had seen a badger-like shape in the thermal imagery. It was a badger. We walked around the hillside to ensure the badger couldn’t smell us in the wind and to take a safe shot. George set up his rifle and Fred got his torch ready. I scarily felt excited. I felt the adrenaline rush through my body. In a hushed voice Fred counted “3,2,1”: he lit the badger up with the torch and George took a shot. Bang, dead. We rushed over to the carcass and put it in a bag (Figure 3). I slung the badger-in-a-bag across my back and carried it for the rest of the night.

My fear and exhilaration shocked and disgusted me as I did not expect, or want, to feel pleasure at death. I was unable to sleep that night. All I could think about was why I experienced such intense emotions in reaction to witnessing the killing of a badger.

### Undertaking Direct Action against the Badger Cull

**FIGURE 4:**
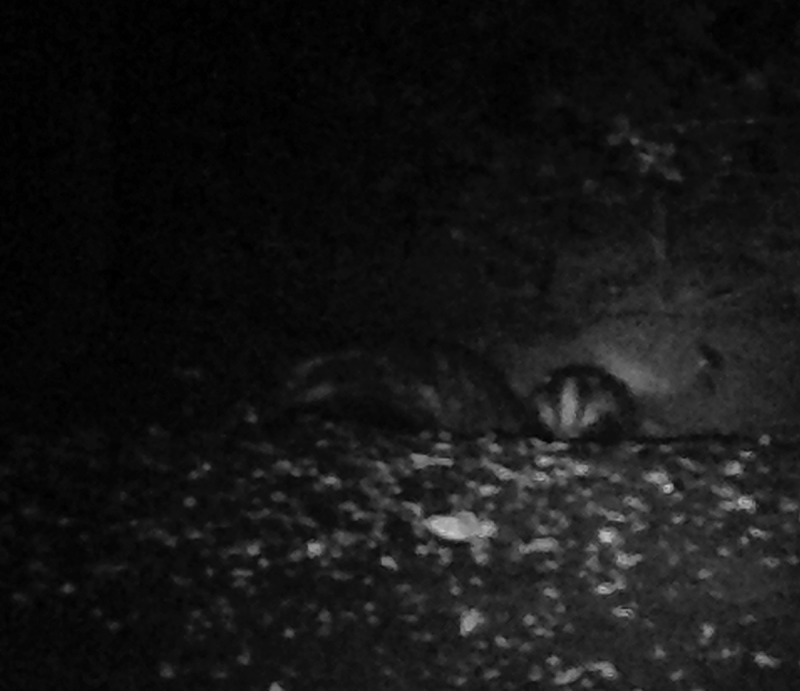
Badgers in the wild. Photo by Jessica Phoenix.

I was with Helen yesterday. We walked and walked on footpaths and shone torches to alert any marksmen of our presence. We must have been walking for five hours and didn’t see any marksmen, or any badgers. I was getting tired when suddenly we heard a rustling and shone our torches in the direction of the sound. There were six badgers in the corner of the field! (Figure 4) What a sight! I stopped still and admired the beautiful creatures for about two seconds before they disappeared into the undergrowth. I strangely felt incredibly protective about their lives.

Like many other people undertaking direct action against the badger cull, Helen self-identified as a “badger protector.” Over a three month period in winter 2016 I undertook participant observation on consecutive nights with people shooting badgers and with badger protectors, sometimes in the same fields. I began to question how I could experience such contradictory emotions between interactions, particularly in light of the spatial and temporal proximity of these encounters.

### Driving Home after a Night Spent Shooting Badgers

I spent last night with Jon [marksman] who shot two badgers. On the drive home, approx. 04:00, I went around a bend and saw a badger in the middle of the road. I slammed on my breaks and managed not to hit it. It scarpered before I could see where it went. I was relieved that I didn’t kill it, but didn’t feel guilty for having been with someone who potentially shot members of its clan earlier in the night. It was like the badgers Jon shot earlier were his responsibility, not mine. Weird.

The next day, I reflected upon this event in my field diary:

The killing instrument of a gun was right, but the killing instrument of a car was wrong. Jon being the killer was right, but me being the killer was wrong. Killing as part of the cull is right, but killing by accident is wrong. Why am I determining the appropriateness of these events through how a badger is killed, my emotions and where it takes place?

### Collecting Badger Carcasses for the Study of bTB

**FIGURE 5:**
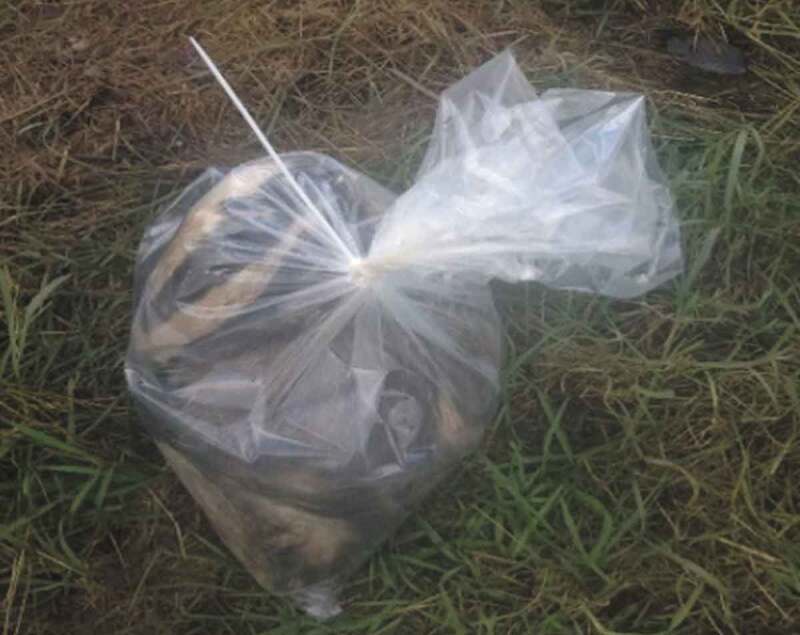
A badger carcass in a collection kit. Photo by Jessica Phoenix.

**FIGURE 6:**
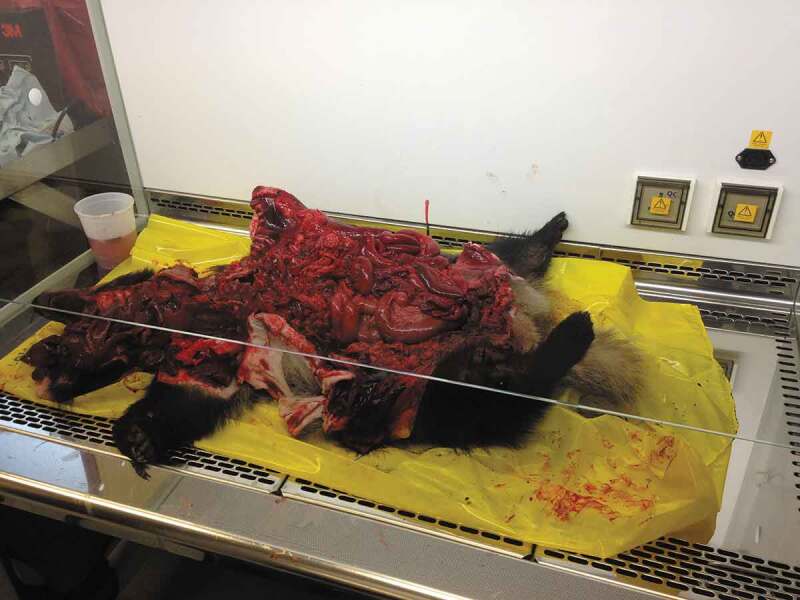
A badger carcass after postmortem. Photo by Jessica Phoenix.

So today I picked up two dead badgers off the roadside [for the badger found dead survey] (Figure 5). On the drive home I considered what they meant to me and I can’t help but think of them as a resource for our research. I didn’t once consider it as a living creature and didn’t consider its pain when it died. All I could think about was not getting rotten badgers on my hands and about conducting a post-mortem (Figure 6). Am I cold hearted?

### Encounters with Badgers

Building on Haraway’s (2008) argument that when and how species meet matters, Hinchliffe (2010, 34) argues that “where species meet can matter, and, […] there might be many wheres involved or folded into species meetings.” Many “wheres” and “hows” folded into my meetings with badgers. As detailed by Wilson (2017) in her review of encounters, each meeting was situated and partial, and therefore invoked a different emotional response, which consequently shaped my relation with the badger that I was co-present with. Meeting a dead badger on the roadside was a different experience to meeting a badger at night in a field. Meeting a badger with someone who had a gun in their hand was a different experience to meeting a badger without. I felt protective, I was surprised and in awe, I considered them to be roadkill and I was excited by the prospect of killing them. These emotions were tied to each how—the people I accompanied and the practices I participated in—and each where—the specific sites, purpose and moments of each encounter.

Hinchliffe suggests that recognizing where species meet should prompt researchers to “intervene at various locations to make for better meetings” (Hinchliffe 2010, 35). However, I felt my ability to make for better meetings was limited by overwhelming and all-encompassing emotions experienced in the moment. In addition, the emotions experienced at particular encounters did not directly correspond to emotions experienced in other encounters. On reflection, I wonder if I somewhat strategically engineered my emotional response in each interaction so that I could perform what I thought was expected of me from the people I was accompanying; such as feeling excited when witnessing a badger being shot when with a shooter and feeling protective when with a badger cull saboteur. The relations I developed with each badger were strongly tied to the contextual framing of badgers as disease carriers, or vermin, or victims of culling (Cassidy 2012). In each moment I therefore developed relations with individual badgers that formed part of, and contributed to, the ongoing polarizing badger culling debates.

My varied experiences with individual badgers throughout my fieldwork helped me to develop more thoughtful relations with badgers as a collective species, not reducible to any single encounter. Over time and away from specific meetings, I became cognizant of interconnections in wheres and, in Hinchliffe’s (2010, 35) words, our collective “fraught geographies.” This does not mean generalizing emotions or actions for all encounters, but rather recognizing the “complex geography of interactions” (Hinchliffe 2010, 35). I still experience excitement when with someone shooting a badger, but I now have a greater capacity to link this with the protectiveness I feel when watching the species with a badger cull saboteur.

My experience of multi-faceted relations with badgers as a collective raises tensions with feminist animal studies scholars who argue that humans should consider animals as individuals, rather than species, so as to consider their well-being (Donovan and Adams 2007; Gruen 2009, 2011; Nussbaum 2006), and to counter speciesism as a form of oppression (Deckha 2011; Harper 2011). This tension is not one I seek to resolve, but rather one I consider as useful to think with. Individual badgers *do* matter, but how I related with each badger drastically differed dependent on each how and where. By focusing only on individual badgers in individual encounters, our relation was immediate, situational and therefore strongly influenced by the purpose of the practice i.e. to kill a badger or to protect a badger. However, stepping away from individual animals and individual settings provided time and space to consider interconnections between these settings, between badgers and between relations. By conducting research in which I purposefully sought to participate in different encounters between humans and badgers, I became more thoughtful to the complexity of these interspecies relations beyond any specific individual encounter.

Such a recognition comes to bear less in ongoing physical encounters, and more where badgers are present in representations—my publications, in policy work, and in public engagement. For example, through my re-presentational work of badgers in this piece I have set up encounters between you, the reader, and badgers. You have met with badgers as beautiful creatures, as a resource for an experiment and as a disease carrier. Like my fieldwork, the complexity of encounters in this piece helps you to develop thoughtful, complex and beyond individual site relations with badgers as a collective. In the midst of a human-wildlife controversy, such thoughtful relations may help less polarized futures to be imagined and created.

## MOVING FURTHER AWAY TO RETURN CLOSER: ON HUMAN-CHICKEN RELATIONS IN THE MULTISPECIES FIELD

### Catherine Oliver, University of Birmingham, UK

When Lacey died, she had been ill for a while: not eating, slow and thin. Tucked up with five other chickens, she fell asleep amongst their warm bodies and didn’t wake up. I wondered when they knew, how they cared for her, how they are different without her. One by one, four more chickens passed away until Primrose was alone, her days spent without her chick-kin in the company of humans, cats, worms, and crows. Drawing from ethnographic field/work with ex-commercial hens, I use this space to think about the distance, mourning, and uncertainty in multispecies research.

A path of “almosts” precedes and follows our movements in the world. Lacey arrived as the biggest, shiniest, bossiest chicken of six. Tentative at first, but quickly confident in her explorations of the coop, yard and under the fence out to the fields, the ditch and through hedgerows, Lacey was beautiful. A nervousness to her new world soon became a comfortable familiarity; Lacey was always first to peck my shoes, demanding more grapes. Until she wasn’t. Shiny feathers, bright eyes and playful mannerisms; the chickens seemed so content that you can forget who we are. These six chickens were rescued from a laying-hen breeder less than a mile from our home. Tipped off on Facebook that the farmer was about to transport them to a farm, my mum dashed to the farm with cat boxes and begged the farmer to let her buy some chickens. A laughing and confused farmer handed over six chickens, indifferent to them as to the thousands of others who pass through.

Caring with and for chickens is, for me, a manifestation of mourning in field/work; mourning is not confined only to those we know but punctuates interspecies encounters. Stanescu (2012, 568) describes this political act of mourning animals when walking through the “meat” supermarket aisle: “This scene overtakes you, and suddenly you tear up. Grief, sadness, and shock overwhelms you, perhaps only for a second. And for a moment you mourn, you mourn for all the nameless animals in front of you.” When we mourn for animals who we do not know, we are marked out as strange. I wonder, does Primrose mourn her chick-kin, ask why she is alone? Fieldwork often requires us to do nothing (Gillespie 2019), to witness and not intervene. The chickens I knew had peaceful deaths only because of human intervention into the violent plans for their lives. How can my care be reconciled with the violent conditions of human supremacy that brought us together?

A theory in and of the flesh (see Davis and Todd 2017; Moraga and Anzaldúa 1981) is borne out of intersubjective, and here interspecies, porosities that bring into sharp focus more-than-human dependencies. The chickens experienced pain in their lives but after their deaths, this pain is shifted from their now peaceful bodies into my own. These multispecies worlds are experienced through an embodied ethic, entangled with but more expansive than mourning. This interspecies relationality of care creates a shared field where my body is not solely me, and not solely my own (Mol 2008). My boundaries and “the field” are semi-permeable, always beyond-human, beyond-chicken, beyond the space we share together.

In ethico-political research, we must both refuse the conditions of the present and propose alternatives. These futures are always constructed within and beyond questions of uncertainty, where “the essentially solitary experience of pain makes the ethical question of how we respond to it politically fraught, since we must deal … with questions about whether suffering is occurring, how it can be understood and whether it is important enough to respond to” (Wadiwel 2016, 2). To care for chickens is not a spectacular grief, but an intentional interruption of the violent human-as-usual that casts a politics of doubt upon the “normal” (animal-eating) world. Uncertainty is not antithetical but rather inherent to radical imaginings of the future, where “the world beyond the human is not a meaningless one made meaningful by humans … rather, mean-ings emerge in a world of living thoughts beyond the human” (Kohn 2013, 72).

As Fusco et al. (2017, 2262) outline, there is a pervasive uncertainty in geographic knowledge production, covering “incomplete knowledge, inaccurate knowledge, imprecise knowledge, fuzzy knowledge, disputed knowledge, ambiguous knowledge, impossible knowledge.” When faced with a seemingly ubiquitous and fixed knowledge of the animals you are with *not* mattering, uncertainty is vital to understanding the impossibility made possible of our more-than-human assemblage. Opening and prioritizing uncertainty thus challenges the way things are, with the ways that things *could* be. Care and attentiveness to the suffering of other-than-human animals does not require absolution, but rather that, even in uncertainty, we know them *well enough* for them to matter (Midgley 1983).

In the multispecies field, our proximal closeness sometimes allows us to breach the obvious somatic distances and difference. In closeness, recurrent and painful histories of violence can infuse the atmospheric space. Being-with chickens in this multispecies field is a realization of a future never meant to be possible. Yet, it remains entangled with a larger mourning echoing through expansive geographies of beyond-human suffering. Primrose’s peace is a mourning denied to billions of others like her, like us. Holding on to uncertainty, foregrounding it, turns us in a vital orientation toward a future beyond the horizon of what and how we know, and who we might become. Rather than presenting solid truths of the way things were and are, we should instead seek “specific material engagements that participate in (re)configuring the world … making knowledge about specific worldly configurations” (Barad 2007, 91).

Caring for animals requires entering another world (Walker 2013), separate but overlapping with our own. This demands openness to beyond-human ethics and politics of mourning and uncertainty. The human interlocutor must attend to the disturbed and disrupted revolutions of everyday life possible when violence in all its forms is refused within and beyond human societies and worlds. Researchers working for and with animals must be urged not only to resist the devastation of humans on beyond-human worlds, but also to imagine alternative futures. Any motif for relation less than this centers us in the reproduction of violence, limits our imagination of the possible and, ultimately, recommits to violence-as-usual.

## TOUCHING THE FOOTPRINTS OF DONKEYS

### Olivia Mason, Durham University, UK

At the entrance to Petra, Jordan, stands one of the site’s many donkeys. He belongs to a young Bedouin boy, who sees me and immediately runs over and asks if I would like a donkey ride. “It’s really far to the Treasury,” he assures me, “and it’s hot … I can take photos—do you have a camera?” He is a good salesman, but I refuse. To me, the donkey looks thin, tired, and has lesions on its neck from the rope cutting into it. I refuse the Bedouin’s offer because these donkey rides are often over-priced and I would rather walk. While the donkey might relieve my body of some of the pain and discomfort of walking, it would merely be transferred to the donkey.

**FIGURE 7:**
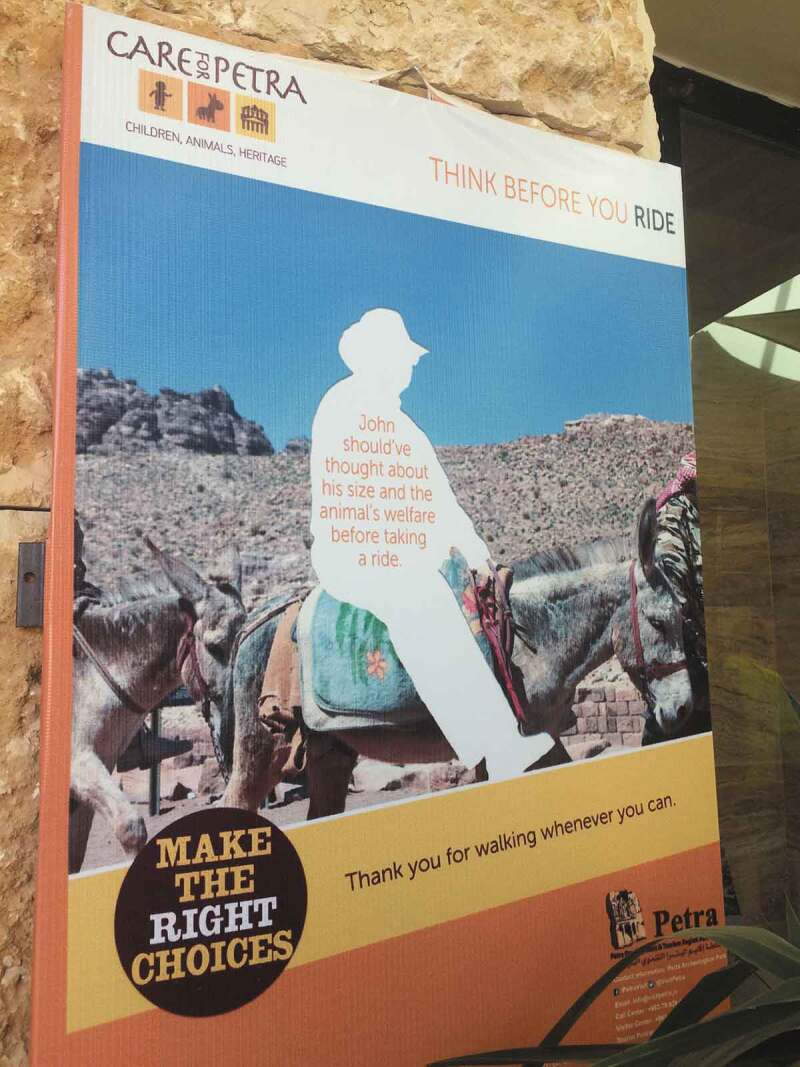
Sign in Petra warning of animal cruelty. Photo by Olivia Mason.

Following a People for the Ethical Treatment of Animals (PETA) (2018) exposé, animal cruelty in Petra has gained broader discussion within Jordan. This includes signs (Figure 7) stating that “the valued tourist” can report any abuse or mistreatment of animals to the Tourist Police alongside responses from the Minister for Tourism and Antiquities and the Royal Family (PETA 2018). In this piece I explore these responses to animal cruelty through the concept of touch. I draw on fieldwork comprised of participant observation and interviews with walkers and Bedouin in Petra in 2016–2017, to argue that understandings of cruelty are shaped through moments of human and more-than-human touch.

Sara Ahmed (1997) argues that touch has a dualistic resonance in that it can refer to the sensation of being affected or moved by something but also the physical contact of one’s body with another surface, the response of receptors in the body to pain, pressure, and temperature. While touching itself does not overcome distance, and, as Ahmed (1997) argues, often emphasizes division, it is always the decision to move forward and interact with something unknown (Manning 2007). Touch therefore requires an ethics of response, a “response-ability,” in which the body’s exposure “to something other than itself” gives meaning to self (Butler 2015, 36). Indeed, as feminist scholars argue, touch is made meaningful through response. Touch and cruelty are brought into relationship in this piece because both the physiological aspects of touch and response-ability to touch are a means by which to explore the cultural politics and power dynamics of animal cruelty in Petra.

**FIGURE 8:**
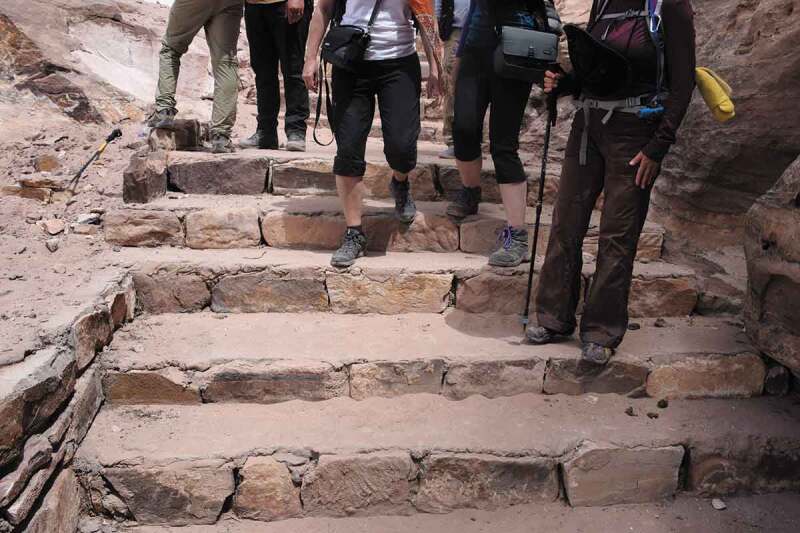
Walkers on the climb to Al-Der. Photo by Olivia Mason.

As I continue my walk, I pass more Bedouin, often young boys, offering donkey rides and see several tourists accepting. I hear a tourist remarking that “no-one told me this would involve so much walking.” Many tourists are not physically able nor prepared for the scale of Petra, especially in the summer heat. From the entrance to one of Petra’s most iconic landmarks, دير ال (Al Der)—The Monastery—the walk is a long, steep climb of just over 3 miles, and donkeys are frequently used to transport tourists. I have done this climb several times, feeling the pain and pressure on my body as I walk—a heightened sense of physiological touch (Figure 8). As I walk past struggling donkeys hauling tourists several times larger, my own experience of pain gives me an appreciation for their labor. It is precisely the strain put on donkeys here that was one of the animal rights abuses highlighted in the PETA report, prompting a response from Jordan’s Prince Alwaheed (Chiorando 2018):

Using animals to carry tourists ruins the beauty and the sanctity of the place. Not because they are animals, but because this hike is something people need to climb themselves and need to experience themselves.

How tourists respond to the pain of the donkeys is shaped by their interactions—how they touch these animals physically and how they are brought into relationality with them. The Prince’s comments suggest that by experiencing the pain of the hike with their own bodies, rather than shifting it onto the body of the animal, the tourists will be more in touch with the sanctity of the landscape. This is conveyed also in the sign in Figure 7, thanking visitors “for walking whenever you can.” Following Kim (2015), who argues that pain is neglected in animals because of lack of knowledge or else when suffering serves a higher purpose, the “higher purpose” of the pain inflicted on animals is questioned by urging individuals to walk and experience the pain of the climb themselves. However, the focus on animal cruelty—as inflected by tourists or Bedouin—obscures the broader politics that shapes the response-ability toward cruelty. It conceals the reality that the use of donkeys in Petra is connected to the commodification of Petra and the postcolonial histories of Jordan.

The designation of Petra as a United Nations Educational, Scientific and Cultural Organization (UNESCO) World Heritage Site in 1985, saw its Bedouin population—the B’doul tribe—displaced and emplaced to a nearby village (Al-Mahadin 2007). Maintaining a Bedouin identity in the representation of the site was crucial, and so the B’doul were given trade permits to encourage their continued presence, forcing them to make new forms of income. While the Bedouin remain present, their displacement has altered their traditional nomadic lifestyle, including the role of animals within it. The universalistic argument used by groups such as PETA, that animal cruelty is wrong because of a universal human sensibility, obscures the situated cultural politics of cruelty (Kim 2015; see also Srinivasan 2013). Further, as Deckha (2008, 37) notes, these discourses are used to “construct (racialized) differences between people and sustain power relations between dominant and marginalized groups.”

**FIGURE 9:**
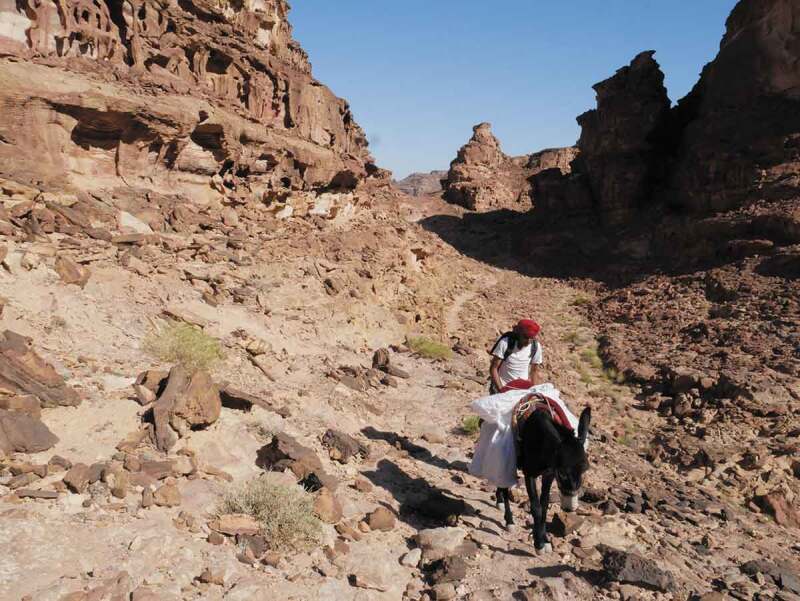
Mahmoud and William Shakespeare just outside Petra. Photo by Olivia Mason.

Helen Wilson (2019, 712) argues that understanding multi-species encounters within postcolonial framings is crucial to raise questions about “knowledge-making, uneven structures of power, and decipherability.” Throughout my fieldwork in Petra, it became evident that human and more-than-human interactions are key to indigenous knowledge making practices. One day on a walk with a group of ex-pat and Jordanian walkers beyond Petra, we were joined by Mahmoud and his donkey—William Shakespeare—who was to carry extra water for us (Figure 9). As well as caring for us, William was cared for by our group. We would stroke his head, offer him water, and feed him bits of our lunch. Mahmoud often found our behavior amusing. That was not to say that Mahmoud did not care for William but instead that his care manifested in a different way. Mahmoud respects William, he trusts him and appreciates the role he plays in carrying water for us and his Bedouin community. Even when Mahmoud is not with William, he follows the footprints of donkeys in the sand. He told me that camels cannot navigate rugged terrain well and goats take routes that are difficult for humans to follow. Donkey footprints are the best to follow, he said; they always know the easiest way through the landscape.

Another day, Mahmoud taught us how Bedouin communicate with goats, sheep and donkeys. Some of the sounds, such as that to make a sheep stop—“rreeei”—are not sounds I could easily make; they require the mouth and the throat to move in ways unfamiliar to my body. The embodied nature of this wordless communication illustrates that animals constantly shape, form, and give meaning to interactions between individuals and space, and that the shape these interactions take differ between indigenous and Western knowledge systems (Haraway 2008; Todd 2018).

For Bedouin, donkeys are not a means of human transportation, but carry water and food and help navigate the rough terrain. Pain is shared. However, the commodification of Petra for tourism and the removal of Bedouin from the site has forced an end to their nomadic lifestyle, and the role animals play within this. While the PETA report draws important attention to the pain of donkeys, it misses the wider cultural, social and political dimensions of animal cruelty in Petra, and remains negligent of non-western relationships with animals. The application of a universalistic lens of cruelty through the report insinuates that pain to animals is only caused through tourist bodies or Bedouin seeking to make economic gain from tourists. It overlooks the culturally situated nature of cruelty, which is shaped by uneven power struggles, commodification, and postcolonialism. Through a consideration of how both tourists and Bedouin are in touch with the pain of donkeys, and a positioning of touch as an ethics of response, a more critical understanding of cruelty in human and more-than-human interactions becomes possible.

## RAINFALL, FELL^3^

### Sarah Thomas, University of Glasgow, UK

What happens when “the field” is your home? What happens when the field floods and the vulnerability of the researcher is not only a source of theoretical consideration but a pressing reality, shared with her neighbours, to which they must co-create a response? What kind of response is appropriate?

**FIGURE 10:**
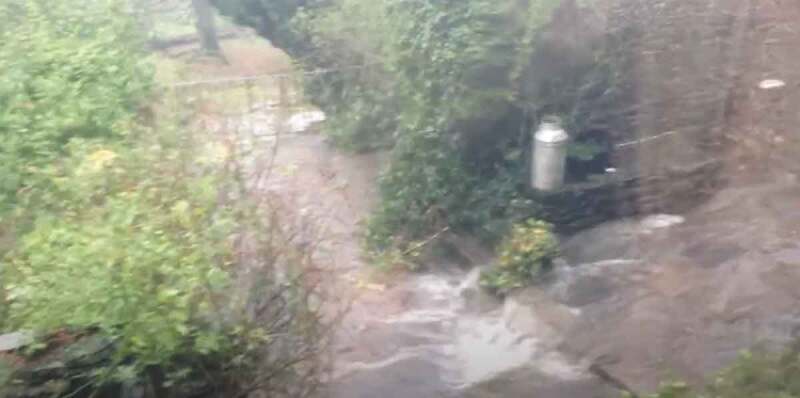
Still from video, taken from my living room window in the early stages of the flood. Link: https://youtu.be/FPyUWK_r8S4 Image by Sarah Thomas.

In December 2015, the Cumbria floods in UK left hundreds without power. Roads, homes and bridges were damaged or destroyed. On the night of the 5th the floodwaters literally washed into my life, leaving the hill on which I live marooned and car travel impossible for a time, resulting in much journeying on foot (Figure 10). A neighbor I had never met was one of the “fatalities” reported in the news. His passing and my walking paved the way for an encounter and subsequent friendship with his widow—a sheep farmer—which profoundly deepened my relationship with the terrain.

The following piece outlines some of the ways in which this relationship with “the field” allowed for a radical interdisciplinarity in my research, drawing together a diversity of voices and narratives and a shared vulnerability which would have been impossible with a model of fieldwork framed by visits. I propose that creative writing is an effective medium through which to communicate the emergent complexity of such sustained relationships in a compelling manner. A creative nonfiction essay *Rainfall, Fell* was my response to this novel situation. Extracts are included below and the full essay is available online.^4^

Creative nonfiction uses literary craft to write affectively about real events and people. Its techniques can be applied to memoir, place writing, life writing, research papers, journal articles and more. *Rainfall, Fell* is set in a specific geographical location in the region where I live—between Kendal and Windermere in England’s “Lake District” and famous sheep farming country. The field is therefore also my home, the “characters” my neighbors, and the journeys taken through the piece are the loops that I walk frequently from my house and back again, in all seasons, with which I weave my own story out of this “unruly tangle of relations.”^5^

**FIGURE 11:**
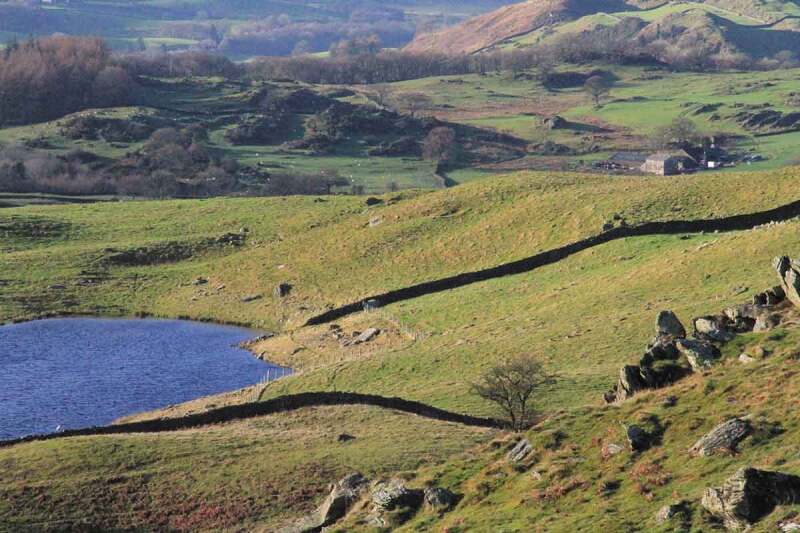
The fell in drier weather. Photo by Sarah Thomas.

**FIGURE 12:**
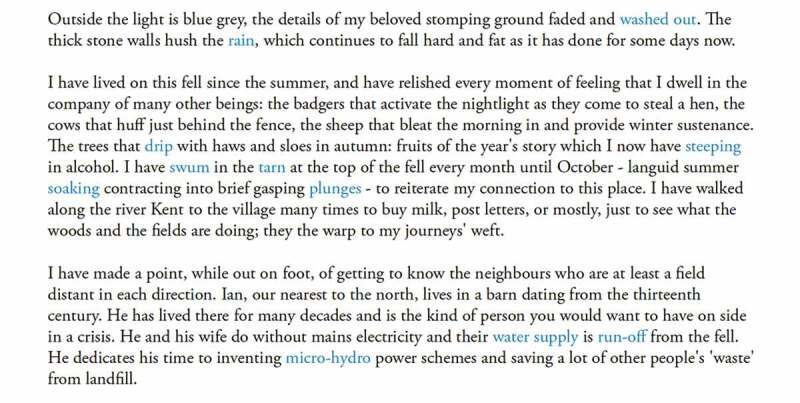
An aqueous vocabulary. Image by Sarah Thomas.

“Fell” is a Cumbrian dialect word for mountain (Figure 11). The title *Rainfall, Fell* is a play on words with “Fell” being noun and verb; place, matter and process; and a metaphor for this web of entanglements. From the position of a narrator on the fell, *Rainfall, Fell* details the night of the floods and their aftermath over the course of the following year, attending to changes in the landscape (the response of the ground, the trees, and the rivers) and the changing human relationships on the fell. It experiments with *languaging* the flows of water through lives and places, and the interconnectedness of those lives (human and other-than-human) through de-centering the human narrator. Such flows are gestured at esthetically as well as linguistically: the text has an aqueous vocabulary running throughout, distinguished in blue (Figure 12).

*Rainfall, Fell* blends memoir and place writing, and layers a diversity of knowledges. Researched and written over a year, reading a variety of texts and walking between my two closest neighbors and up the fell through the seasons, I could weave in real conversations and events in their lives and my own as they unfolded: time was a key ingredient. With conversations had over cups of tea and time given to listening, and relationships characterized by exchanges of help, food and essentials, a recurring motif of mutual aid and hospitality runs through it. It finds hope within the trouble.

While I consulted the National Hydrological Monitoring Programme report and academic papers about the hydrosocial cycle, my participation as a researcher-in-place foregrounded my embodied lived experience in the landscape. By virtue of the field being home, this kind of research then intersected with relevant local and national news articles, Flood Defense Alliance correspondence, community produced ecological surveys, and conversations with local residents. It could incorporate knowledge gained from participating in the cyclical nature of the fell through the prolonged and embedded relationship that dwelling entails: through repeated walking on the fell observing plant and animal life; mucking out a stable over several months; harvesting fruit from fruit trees and sharing what I made with it with neighbors; swimming in the river and the tarn; eating lamb reared on the fell; gathering nuts, plants, berries, and mushrooms in different seasons; speaking with neighbors who had lived there for many decades and observed changes in land use and were aware of the geology, place name etymology and its significance for hydrology and ecology. One was retired Ordnance Surveyor who had mapped the catchment area and now submits daily weather readings to the MET office. Another was the deceased man’s widow whose (now rare) knowledge of Cumbrian dialect was illuminating. An excerpt

“Oh you should see what the people who’ve bought Birkfield have done. It’s stupid.”

“What?”

“Well they wanted that … under floor heating, didn’t they? So they dug the floor down – it was just stone flags on soil – to lay the pipes. And they ended up in 3 foot of water. *Birk* in local dialect means stream. Birk-field … is a field with a stream through it. So you don’t want to be digging below floor level in a house that’s been there just fine since the 1600s.”

**FIGURE 13:**
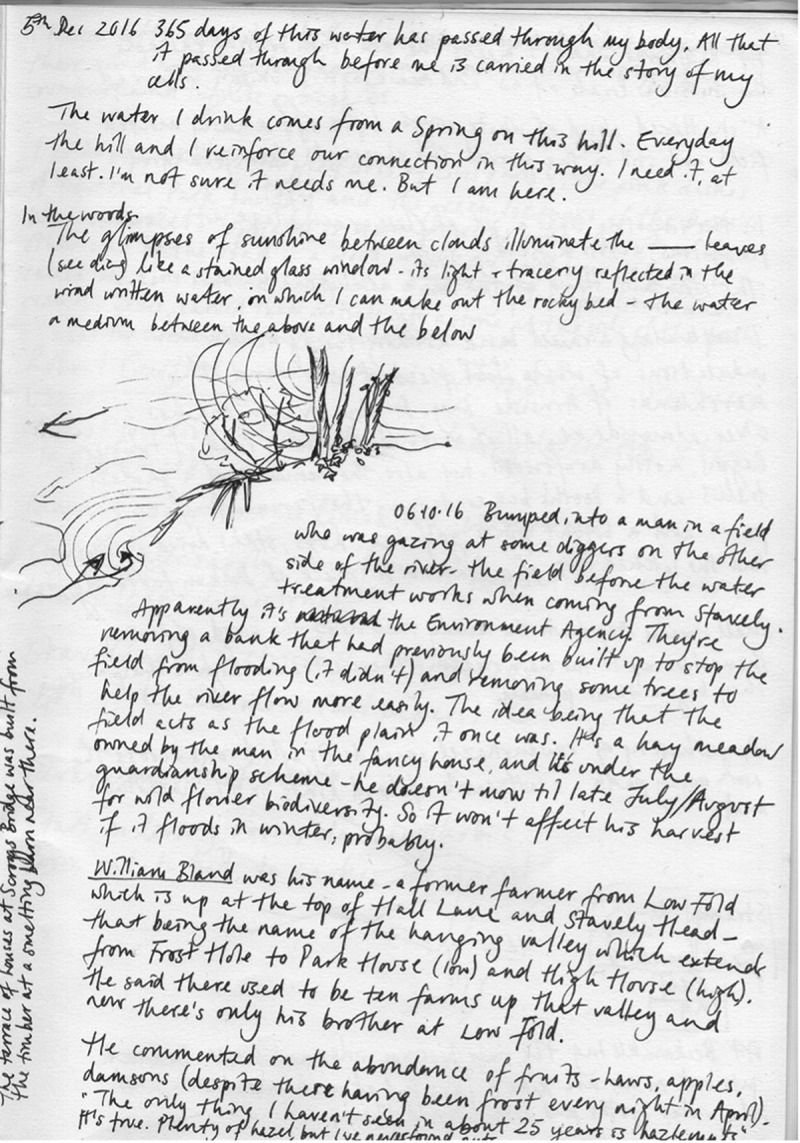
Journal entry. Image by Sarah Thomas.

**FIGURE 14:**
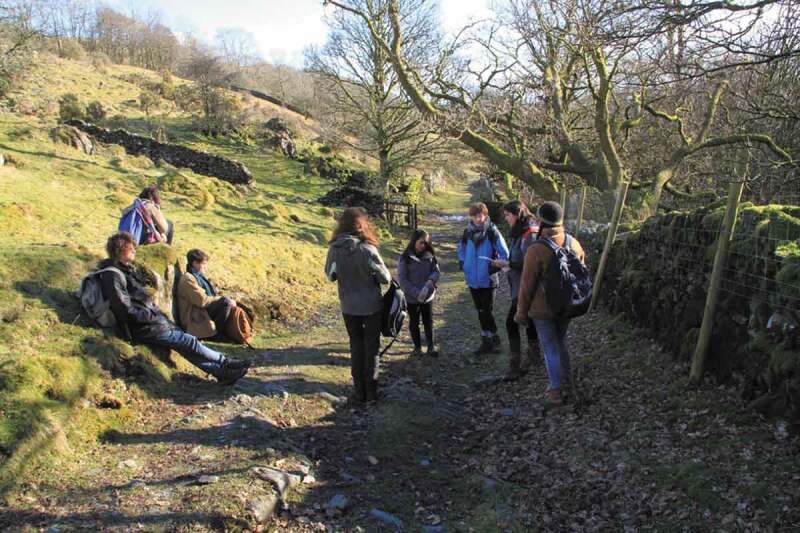
Walking performance of *Rainfall, Fell*. Photo by David Borthwick.

The essay therefore positions local and embodied knowledge as invaluable expertise, giving space on the page to the other-than-human. Attentive to the changing texture of the ground underfoot; to growth and destruction in the landscape, I recorded this slow, quiet observation in a journal (Figure 13). I wrote and read essay drafts in situ outdoors in different seasons. Once complete, I did a walking performance of extracts of *Rainfall, Fell* in different locations on the fell to which it related (Figure 14). I wished the text to be washed through by the flows of water, criss-crossed by my path-making, ripe with hedgerow fruit and tangled with black plastic blown from hay bales up the valley.

To co-create a story—a memorial to this time—which goes beyond the news narratives and the flood reports, which stays around long enough to see what emerges after a crisis, and which digs into the environmental and social history of the place is a way to “take up the unasked for obligation of having met” (Haraway 2016, 130). Such work might be a fitting response to climate events and environmental catastrophe and may be of service to a place’s future: a clear acknowledgment of the entanglements of which we are all a part. To write is to bear witness. To write slowly is to enter into a different kind of time: one which honors the polyrhythms that make a place and holds a space between the situated and the planetary.

Sometimes, life presents a situation or an object which inhabits this space. Another excerpt:

One afternoon in early May, Sylvia^6^ is not outside when I arrive. Instead, she answers the door looking uncharacteristically glum. She guides me to my place on the sofa and sniffs her jumper.

“Sorry. I’ve been scrubbing sheep’s bottoms,” she says with a half-smile.

She disappears into the kitchen and emerges with a cup of tea.

“What’s wrong?” I ask.

“Oh, can you tell?” She perches on the armchair. “It was the inquest the other day. It took me right through it all again.”

She hands me the write-up in the local paper:

*A farmer who died during the December floods was held under a bridge by an oil drum … The hearing was told that he was attempting to clear debris from under the bridge when he became trapped by the steel barrel. Rescue crews could not retrieve his body until the following day, the inquest heard … In the morning of December 7, crews managed to dislodge the oil drum with a pulley system. It flowed from the bridge to a metal gate that had been put in place to stop any potential body from passing. Mr. Woodbridge’s body was still wrapped around the drum as it came loose …* ^7^

“He wasn’t a farmer,” Sylvia huffs. “*I’m* the farmer. They could have got that bit right. People have been coming and snooping around since that. They don’t even say hello!” She stares into the middle distance. “I know I look alright when you come and visit. But it’s the nights I find hard. I put the radio on sometimes but it’s all so *horrible* what’s on there too.”

It feels as if the time has come that she wants to talk about it. But I sense it might come out piecemeal.

“He’d had his porridge and said, ‘We better get out and see about this beck^.^^8^’ We hadn’t done the horses or anything. The horses went without food all day.” She holds a biscuit suspended midway between plate and mouth and tries to keep her composure. “That barrel. It had come down the valley from somewhere. It wasn’t ours.”

A “fatality” which took place where excess rain washed down a deforested valley and met detritus from the fossil fuel industry, and the particularity of Mr. Woodbridge’s untimely end, speaks across time and space to our shared predicament.

## FIELDS SHARED AND REARRANGED: NOTES TO THE READER

### Natalie Marr, Mirjami Lantto, and Maia Larsen, University of Glasgow, UK

We opened the conference session *Visitations: more-than-human field/work encounters* with a string of questions spoken from within the audience, and provocations printed as postcards and left on the seats, our intention being to encourage collective reflection on some of the challenges and propositions that fieldwork stirs up. This compendium article continues in the same spirit. Committed to a critical reflection on fieldwork encounters, and curious where they might take us, we now invite the reader to consider their own experience of sharedness in field/work with more-than-human others: How do bodies become unsettled and rearranged through field encounters? In what ways do non-humans share in our research? How do we thoughtfully attend to the messy, co-constitutive and exposing qualities of fieldwork?

We encouraged the coauthors of this article to start from the encounter—to reflect on process and practice, and how their respective field sites got to work on them (Larsen and Johnson 2016). Read together as a collection, the pieces evoke rupture as much as they do synergy, weaving a questioning, enlivening and sometimes unsettling conversation that retains the particularities of the authors’ respective pieces. With each piece, the reader is situated anew, prompted to think with the traces of these encounters, and the tensions and incommensurabilities that this involves. Questions and challenges raised in one account may find further expression and expansion in another, but equally, complexity and conflict. There is much that remains unresolved.

Hesitations and tensions within and between the pieces evoke the challenge of encountering others, both in our fieldwork and in our writing. In coming together and pulling apart, the vignettes compose an account of the varied experiences of sharedness in fieldwork, conjuring a collective sense of the uncertainty, vulnerability, and estrangement that it entails.

Sharedness is explored as ethical ambiguity, insofar as several vignettes involve reflection on our uneasy relations with animals, and the different ways we attend to, and contribute to, their suffering. Shifting between the lives and deaths of battery hens and badgers, for instance, involves a change of tone that leaves us unsettled in our ethical responsibility toward non-human others.

Sharedness as generative vulnerability and exposure is brought to bear in, for instance, troubling moments of touch in Petra’s tourist trails, and at the site of bodily exchange between researcher and mosquito in the mangroves of Sydney’s Georges River. While distressing, harmful exposures also emerge as a way of tentative knowing between species.

Sharedness is also intimated in acts of care-ful attention to our wider ecologies, through the compositional drawing of transient and multiple crane bodies, and through slower, embedded forms of attention, place-relation and languaging in the wake of a flood. Here, to share is not so much to have and to hold, but to bear witness to what escapes our full understanding, to become capable of response.

Through these collected vignettes of more-than-human fieldwork encounters, this article offers reflections on shared realities that become rearranged through cellular attunements, porous corporealities, fraught multispecies relations, and the entanglements of the situated and the planetary. The authors describe shared realities that feel both immediate and estranging, hopeful and troubling, entangled and uneven. Through reflective and affective accounts of field/work, we are encouraged to “think with and from the contingencies of the field” (Buchanan, Bastian, and Chrulew 2018, 387) and to explore what this might entail, as we are fielded into troubled and promising rearrangements of shared futures.

## Notes

1. This research discusses the effect of *Wolbachia* on viruses similar to RRV.

2. I have used pseudonyms for all research participants throughout to preserve participants’ anonymity.

3. “Fell” is the Cumbrian dialect word for mountain.

^4.^
*Rainfall, Fell* is available online here: https://www.yumpu.com/en/document/read/56750122/realistic-utopias-writing-for-change/4.

^5.^
Wording from call for papers: “Visitations: more-than-human field/work encounters,” RGS-IBG Annual Conference 2019.

^6.^
I have used pseudonyms throughout this piece to protect the identities of the people I describe.

^7.^
*Westmoreland Gazette*, May 2015. The details of this publication are minimal so as to protect the identities of those involved.

^8.^
^“^Beck” is the Cumbrian dialect word for stream.
